# Perioperative Chemotherapy in Bladder and Upper Tract Urothelial Carcinoma: Outcomes by Nodal Status and Lymphovascular Invasion

**DOI:** 10.3390/cancers17243986

**Published:** 2025-12-14

**Authors:** Nobuki Furubayashi, Jiro Tsujita, Azusa Takayama, Yuta Shiraishi, Motonobu Nakamura, Takahito Negishi

**Affiliations:** Department of Urology, National Hospital Organization Kyushu Cancer Center, Notame 3-1-1, Minami-ku, Fukuoka 811-1395, Japan

**Keywords:** urothelial carcinoma, bladder cancer, upper tract urothelial carcinoma, neoadjuvant chemotherapy, adjuvant chemotherapy, lymphovascular invasion

## Abstract

**Background/Objectives:** Optimal selection for perioperative therapy in urothelial carcinoma (UC) remains uncertain. We evaluated the efficacy of neoadjuvant and/or adjuvant chemotherapy (NAC/AC) for patients with bladder cancer (BC) and upper tract UC (UTUC), examined the role of lymphovascular invasion (LVI), and considered the implications for adjuvant nivolumab. **Methods:** We retrospectively analyzed consecutive patients who underwent radical cystectomy or radical nephroureterectomy at a single center (July 1998–April 2021; observation to 31 March 2025). After exclusions, 252 BC and 153 UTUC patients were included. Endpoints were cancer-specific survival, progression-free survival (PFS; BC), non-urinary-tract recurrence-free survival (NUTRFS; UTUC), and overall survival (OS). Survival was estimated by Kaplan–Meier analysis and compared by log-rank tests. **Results:** For BC, AC did not improve the PFS or OS in the overall pT ≥ 2 population, whereas node-positive (pN+) disease derived significant benefits in both endpoints among NAC-naïve patients (PFS and OS, *p* = 0.002 and *p* = 0.008). For UTUC, AC conferred no advantage in NUTRFS or OS for the overall pT ≥ 2 population. However, NUTRFS benefits emerged in the pN+ subset (*p* = 0.049), although the OS was not improved. Among NAC-treated BC, the outcomes were poorest for ≥ypT3 and ypN+, whereas ypT ≤ 2 fared better. LVI was associated with adverse outcomes and was borderline higher in pN+ versus pT ≥ 2/pN− for BC (*p* = 0.056) and significantly higher for UTUC (*p* = 0.012). **Conclusions:** In this retrospective, single-center cohort, our exploratory analyses suggest that perioperative benefit is largely node-dependent, supporting prioritizing systemic therapy for pN+ disease and cautioning against routine AC for pT2/ypT2 without nodal involvement. After NAC, adjuvant therapy appeared most justified for ≥ypT3/ypN+. Prospective biomarker-integrated validation is warranted and, given the small and underpowered subgroups and the potential for selection and immortal time biases, these observations should be interpreted as hypothesis-generating rather than causal.

## Simple Summary

Urothelial carcinoma (UC) is often treated with radical surgery, yet relapses often occur. Currently, perioperative systemic therapy is used to lower the risk of recurrence. We reviewed the outcomes of patients from a single cancer center who underwent radical cystectomy for bladder cancer and radical nephroureterectomy for upper tract UC. Adjuvant chemotherapy did not improve the survival of patients with organ-confined, node-negative disease, but it was associated with benefits in those with node-positive disease. In the upper tract cohort, adjuvant chemotherapy only improved non-urinary-tract recurrence-free survival in node-positive patients. Among patients who received neoadjuvant chemotherapy, postoperative risk was concentrated in ≥ypT3 or ypN+ subgroups. Lymphovascular invasion was linked to biological aggressiveness and nodal involvement. Overall, these findings support a node-directed approach to adjuvant therapy and suggest that routine treatment of pT2/ypT2 without nodal disease may risk overtreatment, underscoring the importance of careful patient selection and prospective validation.

## 1. Introduction

Urothelial carcinoma (UC) remains a challenging malignancy, with substantial risks of recurrence and progression despite advances in multimodal care. For patients without distant metastases, definitive surgery is a cornerstone of curative-intent management—radical cystectomy for muscle-invasive bladder cancer (MIBC) and radical nephroureterectomy for high-risk upper tract urothelial carcinoma (UTUC)—as recommended by contemporary international guidelines [1,2,3,4,5,6]. In addition, perioperative systemic therapy has long been used to improve oncologic outcomes.

In MIBC, contemporary guidelines recommend cisplatin-based neoadjuvant chemotherapy (NAC) for cisplatin-eligible patients, and an individual-patient meta-analysis of randomized trials demonstrated a significant overall-survival benefit with platinum-combination NAC (hazard ratio [HR] 0.86, 95% confidence interval [CI] 0.77–0.95), corresponding to an ~5% absolute increase in the 5-year overall survival (OS) and an ~9% absolute gain in 5-year disease-free survival [1,3,7]. In UTUC, the randomized phase 3 POUT trial established adjuvant platinum-based chemotherapy after radical nephroureterectomy as the standard of care, demonstrating a significant improvement in disease-free survival (HR 0.55, 95% CI 0.38–0.80), corresponding to an ~17% absolute increase in 5-year disease-free survival (62% vs. 45%) and a favorable trend in OS (5-year OS 66% vs. 57%; HR 0.68, 95% CI 0.46–1.00) [8,9].

In metastatic settings, the EV-302 trial showed that enfortumab vedotin plus pembrolizumab significantly outperformed platinum chemotherapy (median OS 31.5 vs. 16.1 months), with a confirmed objective response rate of ~68% and 29–30% complete responses. Although follow-up now extends to approximately 2.5 years, the median duration of response was not reached at the primary analysis, and durable complete remissions appear confined to a subset of patients [10,11]. These observations underscore that for potentially curable patients, preventing relapse through optimized perioperative strategies and refined patient selection remains a central objective. Against this backdrop, adjuvant nivolumab has emerged as a perioperative immunotherapy option after radical surgery for high-risk UC. In the CheckMate 274 trial, adjuvant nivolumab significantly improved disease-free survival versus placebo in patients meeting high-risk criteria (no NAC: pT3–4 and/or pN+; with NAC: ypT2–4 and/or ypN+) [12]. Nivolumab has since been approved for the adjuvant treatment of high-risk UC in multiple regions, including Japan.

Given the residual risk of recurrence despite contemporary systemic strategies, refining pathological risk stratification is crucial to guide perioperative therapy. Lymphovascular invasion (LVI) is a robust adverse pathological feature across UC. Meta-analyses show that LVI leads to worse outcomes after radical surgery in bladder cancer and UTUC, and several retrospective studies have suggested that adjuvant chemotherapy may preferentially benefit LVI-positive UTUC [13,14,15,16]. However, analyses of bladder cancer have not consistently confirmed predictive interactions between LVI status and the benefit of adjuvant chemotherapy, underscoring the need for prospective validation [13,17].

Accordingly, we evaluated perioperative treatment outcomes (neoadjuvant and adjuvant chemotherapy) in institutional cohorts of bladder cancer and UTUC, reassessed patient eligibility under current adjuvant nivolumab criteria, and explored whether LVI is associated with differential benefits from adjuvant chemotherapy. Because our analyses are retrospective and involve several small subgroups, they are intended as exploratory and hypothesis-generating to inform future biomarker-integrated prospective studies rather than to provide definitive treatment directives.

## 2. Patients and Methods

### 2.1. Patient Population

We retrospectively evaluated 434 patients with localized or locally advanced carcinoma of the upper or lower urinary tract without distant metastasis (M0) who underwent radical cystectomy (RC, *n* = 276) or radical nephroureterectomy (RNU, *n* = 158) at Kyushu Cancer Center (July 1998–April 2022). The observation period spanned July 1998 to March 2025 (data cutoff, 31 March 2025). Because adjuvant nivolumab was approved and became available in Japan in April 2022, we restricted the surgical cohort to procedures performed on or before March 2022. Twenty-nine patients were excluded because of the absence of any urothelial carcinoma component, insufficient clinical data, or concomitant RC and RNU performed during the same admission period. Consequently, 405 patients were included in the final analysis: 252 with bladder cancer (BC) and 153 with upper tract urothelial carcinoma (UTUC). Of the 153 UTUC patients, 8 (5.2%) received NAC. Because NAC was rarely administered in UTUC at our institution and could confound stage-stratified postoperative analyses, these eight patients were excluded from the primary UTUC cohort, leaving 142 NAC-untreated UTUC patients for the main analyses.

The following variables were collected and analyzed: age, sex, tumor site, histologic subtype, receipt of neoadjuvant or adjuvant chemotherapy, pathological stage, lymphovascular invasion (LVI) status, progression-free survival (PFS; for UTUC, non-urinary tract recurrence-free survival [NUTRFS]), and overall survival (OS). Tumor stage was assigned according to the 8th edition of the TNM classification [18]. Patients with pathological node-negative disease (pN0) were stratified by pathological T stage (pT), whereas those with nodal metastasis (pN+) were classified into the pN+ group irrespective of pT.

### 2.2. Selection of Platinum-Based Neoadjuvant or Adjuvant Chemotherapy

At our institution, the general indications for neoadjuvant chemotherapy (NAC) during the study period were muscle-invasive bladder cancer (MIBC; ≥pT2) diagnosed by transurethral resection of bladder tumor (TURBT) or clinically positive lymph nodes (cN+) identified by computed tomography (CT) or magnetic resonance imaging (MRI). Adjuvant chemotherapy (AC) was considered for patients with MIBC who did not receive NAC and had pathological stage ≥pT2 or pN+ disease based on the RC specimen. Generally, NAC was not administered to patients with UTUC. Indications for AC in UTUC included pathological stage ≥pT3 or pN+ based on the RNU specimen.

The selection of chemotherapy regimens and the number of cycles administered for NAC or AC were determined by the treating physician, based on agents approved for insurance coverage during each period. The available regimens included methotrexate, vinblastine, doxorubicin, and cisplatin (MVAC); gemcitabine plus cisplatin (GC); and gemcitabine plus carboplatin (GCar). The cisplatin dose was adjusted at the physician’s discretion according to each patient’s general condition, including age, renal function, and performance status.

### 2.3. Ethics Statement

This retrospective study was conducted in accordance with the ethical standards of the Declaration of Helsinki and was approved by the ethics review board of the National Hospital Organization Kyushu Cancer Center (authorization no. 2014-99). In accordance with the guidelines of the ethics committee and national ethical standards in Japan, written informed consent was not required, as this was a retrospective and/or observational study using existing materials, such as medical records and documents (opt-out approach).

### 2.4. Statistical Analysis

All statistical procedures were conducted using EZR (version 1.68; Saitama Medical Center, Jichi Medical University, Saitama, Japan) [19]. Continuous variables were compared using the Mann–Whitney U test, and categorical variables were compared using Fisher’s exact test (two-sided). Non-parametric tests were selected because age and other continuous variables did not follow a normal distribution.

Cancer-specific survival (CSS) for BC and UTUC was defined as the time from RC or RNU to death from urothelial carcinoma or the last follow-up. For BC, progression-free survival (PFS) was defined as the time from RC to recurrence or death from the disease. For UTUC, non-urinary tract recurrence-free survival (NUTRFS) was used instead of PFS, considering the distinct recurrence patterns associated with upper tract disease. NUTRFS was defined as the time from RNU to recurrence outside the urinary tract or death from the disease. Survival outcomes were estimated using the Kaplan–Meier method. Patients without an event were censored at the date of the last follow-up. Differences between subgroups were compared using the log-rank test. A two-sided *p*-value < 0.05 was considered statistically significant. Analyses were conducted on an available-case (complete-case) basis; patients with missing data for a given variable were excluded from analyses involving that variable.

## 3. Results

### 3.1. Patient Characteristics (Table 1)

#### 3.1.1. Bladder Cancer (*n* = 252)

NAC was administered to 57 patients (22.6%) who were younger than those undergoing RC alone (median 66 vs. 71 years, *p* = 0.002) but with a similar sex distribution (75.4% male in both groups). Pure urothelial histology was less frequent after NAC than after RC alone (73.7% vs. 91.8%, *p* = 0.001). Pathologic stage distributions were comparable between RC and NAC-RC (≤pT1 42.1%, pT2 17.1%, ≥pT3 28.6%, pN+ 12.3%). LVI was present in 33.3% of overall BC patients and did not differ by NAC status. AC was administered to 56 patients (22.2%).

**Table 1:** Baseline patient characteristics of the overall cohort and stratified bladder cancer and upper tract urothelial carcinoma cohorts.

Characteristics		Bladder Cancer		*p* Value		Upper Tract Urothelial Carcinoma		*p* Value
	Total	RC	NAC-RC		Total	RNU	RNU-AC	
	*n* = 252	*n* = 195	*n* = 57		*n* = 142	*n* = 113	*n* = 29	
Age (years), median (IQR)	70 (63–77)	71 (65–77)	66 (62–72)	0.002	72 (65–77)	74 (66–79)	69 (61–72)	0.002
Male sex, *n* (%)	190 (75.4)	147 (75.4)	43 (75.4)	>0.999	102 (71.8)	80 (70.8)	22 (75.9)	0.651
Pure UC in histologic testing, *n* (%)	221 (87.7)	179 (91.8)	42 (73.7)	0.001	123 (86.6)	13 (11.5)	6 (20.7)	0.223
Neoadjuvant Chemotherapy	57 (22.6)	-	57 (100)	<0.001	-	-	-	-
pT (ypT), *n* (%)				0.623				<0.001
≤pT1 (ypT1)	106 (42.1)	82 (42.1)	24 (42.1)		62 (43.7)	60 (53.1)	2 (6.9)	
pT2 (ypT2)	43 (17.1)	36 (18.5)	7 (12.3)		25 (17.6)	21 (18.6)	4 (13.8)	
≥pT3 (ypT3)	72 (28.6)	55 (28.3)	17 (29.8)		42 (29.6)	25 (22.1)	17 (58.6)	
pN+ (ypN+)	31 (12.3)	22 (11.3)	9 (15.8)		13 (9.2)	7 (6.2)	6 20.7)	
LVI positive, *n* (%)	84 (33.3)	65 (33.3)	19 (33.3)	>0.999	38 (27.0)	21 (18.8)	17 (58.6)	<0.001
Adjuvant chemotherapy	56 (22.2)	42 (21.5)	14 (24.6)	0.717	29 (20.4)	0	29 (100)	<0.001

IQR, interquartile range; UC, urothelial carcinoma; y, yeled; LVI, lymphovascular invasion; RC, radical cystectomy; NAC, neoadjuvant chemotherapy; RNU; radaical nephroureterectomy; AC, adjuvant chemotherapy.

#### 3.1.2. Upper Tract UC (*n* = 142)

Patients receiving postoperative AC were younger than those with surgery alone (median 69 vs. 74 years, *p* = 0.002) and the sex distribution and pure UC rates were similar. Pathology differed between RNU and RNU-AC (≤pT1 53.1% vs. 6.9%, pT2 18.6% vs. 13.8%, ≥pT3 22.1% vs. 58.6%; pN+ 6.2% vs. 20.7%; all *p* < 0.001). LVI was more frequent with RNU-AC (58.6% vs. 18.8%, *p* < 0.001). By definition, AC use was 0% for RNU and 100% for RNU-AC.

### 3.2. Efficacy of Neoadjuvant and Adjuvant Chemotherapy in the Bladder Cancer Cohort: Lymphovascular Invasion by Adjuvant Chemotherapy Status

Among the 252 patients in the bladder cancer cohort, 57 (22.6%) received neoadjuvant chemotherapy (NAC), whereas 195 (77.4%) did not. In the NAC-untreated group, pathological staging after radical cystectomy showed that 82 patients (42.1%) were tumor stage <pT2, 36 (18.5%) were pT2, and 55 (28.2%) were ≥pT3 without lymph node metastasis (pN−). An additional 22 patients (11.3%) had lymph node metastasis (pN+) (Table 1).

**Figure 1:**
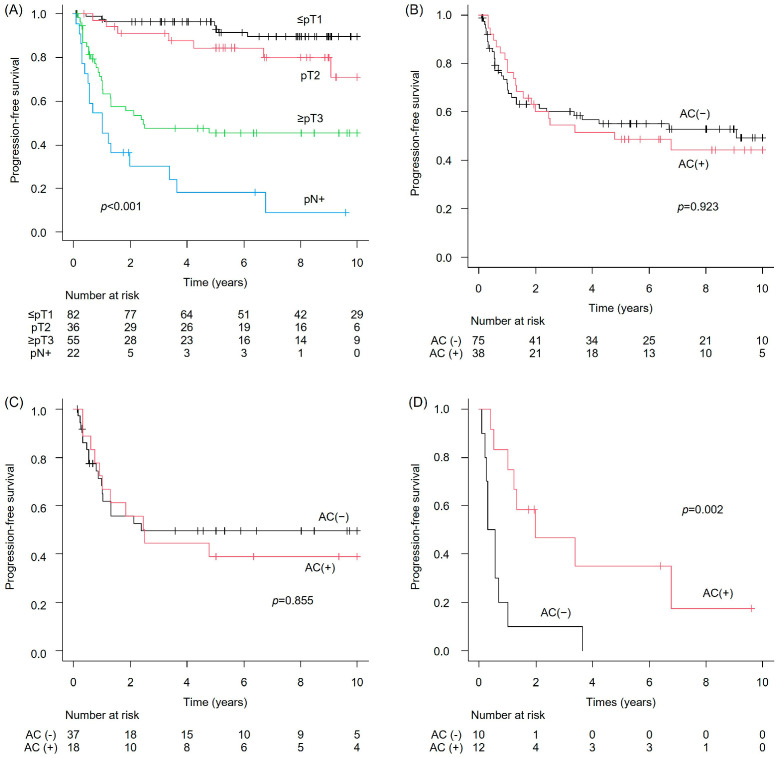
Progression-free survival (PFS) in the neoadjuvant chemotherapy (NAC)-untreated bladder cancer cohort. (**A**) Overall cohort; (**B**) pT2 with versus without adjuvant chemotherapy (AC); (**C**) ≥pT3 with versus without AC; and (**D**) pN+ with versus without AC.

**Figure 2:**
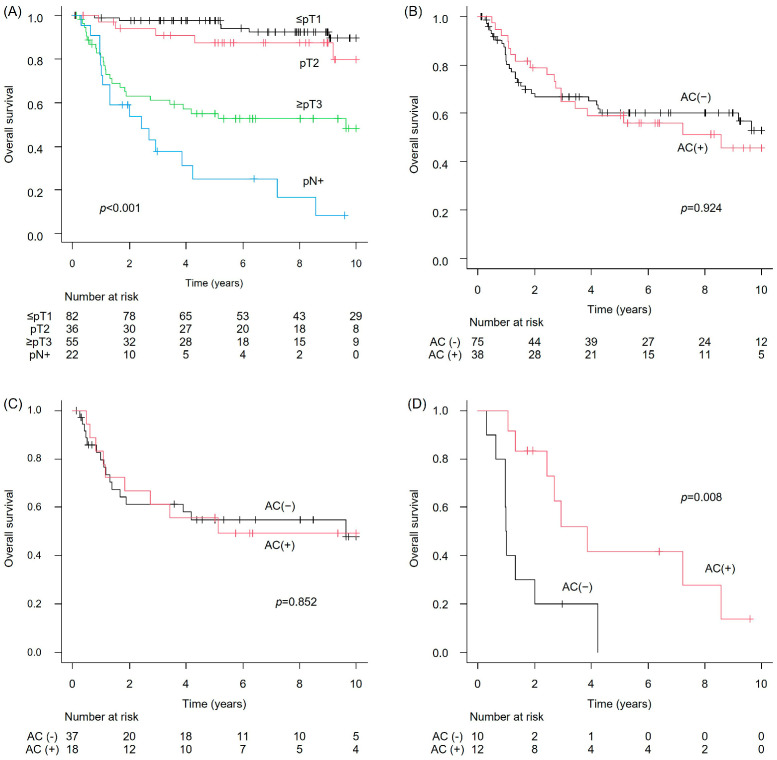
Overall survival (OS) in the NAC-untreated bladder cancer cohort. (**A**) Overall cohort; (**B**) pT2 with versus without AC; (**C**) ≥pT3 with versus without AC; and (**D**) pN+ with versus without AC.

Progression-free survival (PFS) in the NAC-untreated group showed that the median PFS was not reached (NR) in patients with ≤pT1 (95% CI: not estimable [NE]–NE), NR in those with pT2 (95% CI: 9.1–NE), 2.5 years in those with ≥pT3 (95% CI: 1.0–NE), and 1.0 year in patients with pN+ (95% CI: 0.39–3.4). These differences were statistically significant (*p* < 0.001) (Figure 1A). When comparing PFS between patients who received adjuvant chemotherapy (AC) and those who did not, no significant difference was observed in the pT2 or ≥pT3 subgroups (*p* = 0.923 and 0.855, respectively) (Figure 1B,C). However, in the pN + subgroup, the PFS was significantly better for patients who received AC than for those who did not (*p* = 0.002) (Figure 1D). Regarding overall survival (OS) in the NAC-untreated group, the median OS was NR in the ≤pT1 group (95% CI: NE–NE), NR in the pT2 group (95% CI: 10.2–NE), 9.6 years in the ≥pT3 group (95% CI: 1.9–NE), and 2.4 years in the pN+ group (95% CI: 1.0–4.2). These differences were statistically significant (*p* < 0.001) (Figure 2A). Consistent with the PFS findings, AC was not associated with an improvement in OS in the pT2 or ≥pT3 subgroups (*p* = 0.942 and 0.852, respectively), whereas a significant benefit in OS was observed in the pN+ group (*p* = 0.008) (Figure 2B–D). The incidence of LVI did not differ significantly between pT ≥ 2/pN− disease (44/91; 48.4%) and pN+ disease irrespective of pathological T stage (16/22; 72.7%) (*p* = 0.056).

**Figure 3:**
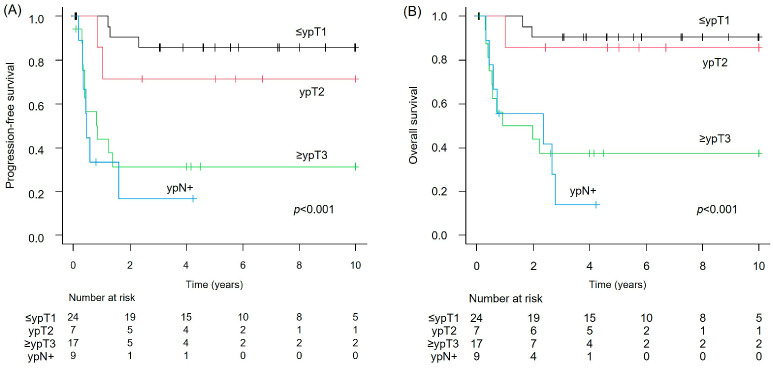
NAC-treated bladder cancer cohort. (**A**) PFS by pathological T stage and nodal status; and (**B**) OS by pathological T stage and nodal status.

In the NAC-treated group (*n* = 57), pathological evaluation showed that 24 patients (42.1%) had ≤ypT1, 7 (12.3%) had ypT2, and 17 (29.8%) had ≥ypT3 without lymph node metastasis (ypN−). Additionally, nine patients (15.8%) had lymph node metastasis (ypN+). In this group, PFS differed significantly by pathological stage: the median PFS was NR for patients with ≤ypT1 (95% CI: NE–NE), NR for ypT2 (95% CI: 0.8–NE), 0.8 years for ≥ypT3 (95% CI: 0.3–NE), and 0.5 years for ypN+ (95% CI: 0.2–1.6) (*p* < 0.001) (Figure 3A). The OS analysis in the NAC-treated cohort showed NR for ≤ypT1 (95% CI: NE–NE), NR for ypT2 (95% CI: 1.0–NE), 1.4 years for ≥ypT3 (95% CI: 0.4–NE), and 2.3 years for ypN+ (95% CI: 0.3–2.8), with a statistically significant difference between these subgroups (*p* < 0.001) (Figure 3B).

### 3.3. Efficacy of Adjuvant Chemotherapy in the Upper Urinary Tract Urothelial Carcinoma Cohort: Lymphovascular Invasion by Adjuvant Chemotherapy Status

Among the 150 patients in the upper tract urothelial carcinoma (UTUC) cohort, 8 (5.3%) received NAC, whereas 142 (94.7%) did not. In the NAC-untreated group, pathological staging after radical nephroureterectomy showed that 62 patients (43.7%) had tumor stage <pT2, 25 (17.6%) had pT2, and 42 (29.6%) had ≥pT3, all without lymph node metastasis (pN−). Lymph node metastasis (pN+) was observed in 13 patients (9.2%) (Table 1).

**Figure 4:**
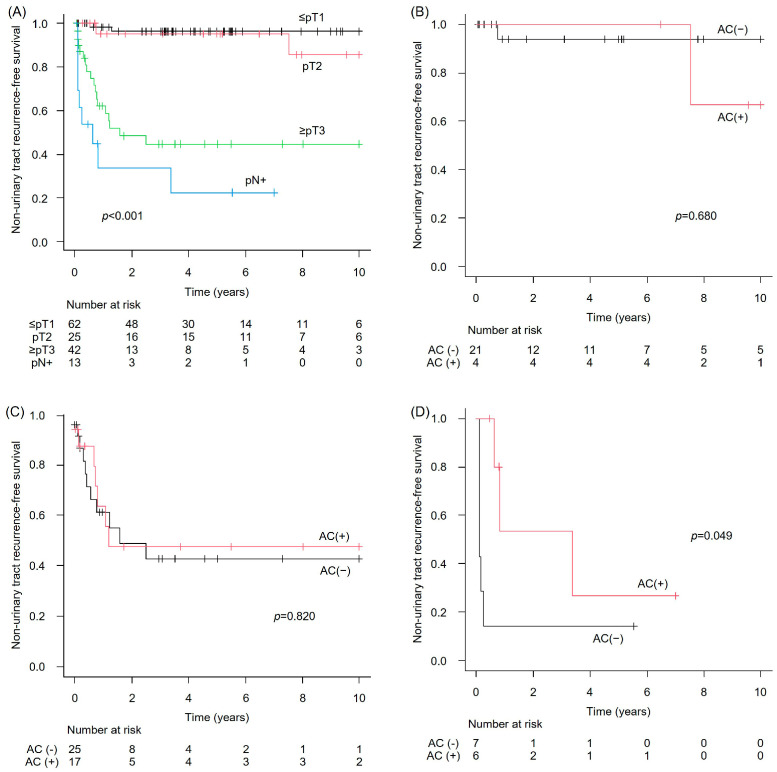
Non-urinary-tract recurrence-free survival (NUTRFS) in the NAC-untreated upper tract urothelial carcinoma cohort. (**A**) Overall cohort; (**B**) pT2 with versus without AC; (**C**) ≥pT3 with versus without AC; and (**D**) pN+ with versus without AC.

Non-urinary tract recurrence-free survival (NUTRFS) in the NAC-untreated group showed that the median PFS was NR in patients with ≤pT1 (95% CI: NE–NE), NR in those with pT2 (95% CI: 10.4–NE), 1.6 years in those with ≥pT3 (95% CI: 0.8–NE), and 0.6 years in patients with pN+ (95% CI: 0.1–NE). These differences were statistically significant (*p* < 0.001) (Figure 4A). When comparing NUTRFS between patients who received AC and those who did not, no significant difference was observed in the pT2 or the ≥pT3 subgroups (*p* = 0.680 and 0.855, respectively) (Figure 4B,C). However, in the pN+ subgroup, PFS was significantly better in patients who received AC than in those who did not (*p* = 0.049) (Figure 4D).

**Figure 5:**
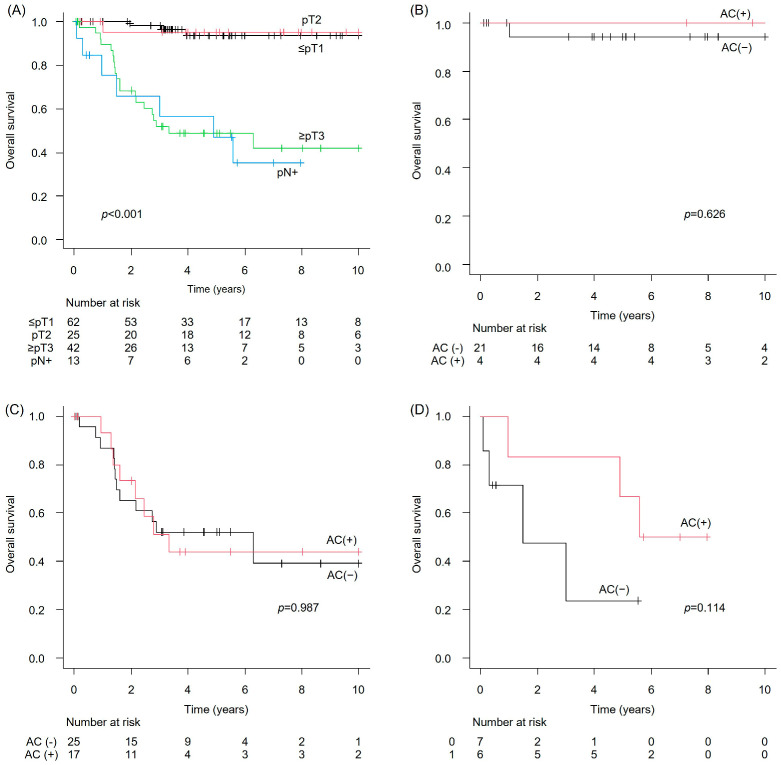
Overall survival (OS) in the NAC-untreated upper tract urothelial carcinoma cohort. (**A**) Overall cohort; (**B**) pT2 with versus without AC; (**C**) ≥pT3 with versus without AC; and (**D**) pN+ with versus without AC.

Regarding OS in the NAC-untreated group, the median OS was NR in the ≤pT1 group (95% CI: NE–NE), NR in the pT2 group (95% CI: 13.1–NE), 3.3 years in the ≥pT3 group (95% CI: 2.2–NE), and 4.9 years in the pN+ group (95% CI: 1.0–NE). These differences were statistically significant (*p* < 0.001) (Figure 5A). AC was not associated with a significant improvement in OS in the pT2 or the ≥pT3 subgroups (*p* = 0.626 and 0.987, respectively) or in the pN+ group (*p* = 0.114) (Figure 5B–D).

The incidence of LVI differed significantly between patients with pT ≥ 2/pN− disease (24/67) and those with pN+ disease, irrespective of pT (10/13) (*p* = 0.012).

## 4. Discussion

This retrospective study evaluated the efficacy of perioperative chemotherapy (neoadjuvant and/or adjuvant) in patients undergoing radical cystectomy for bladder cancer or radical nephroureterectomy for upper tract urothelial carcinoma, with particular attention paid to LVI status. Our results show that for BC, AC did not improve PFS or OS in patients with pT ≥ 2, whereas pN+ disease derived significant gains in both endpoints among NAC-naïve patients. For UTUC, AC did not confer a significant advantage in NUTRFS or OS in patients with pT ≥ 2, but a PFS benefit emerged in the pN+ subset among NAC-naïve patients. In the NAC-naïve cohorts of BC and UTUC, the incidence of LVI differed significantly between those with pT ≥ 2/pN− or pN+ disease. Taken together, these LVI patterns suggest that AC might have greater benefit for pN+ patients. In addition, in the NAC-treated BC cohort, the PFS and OS were worse for patients with ≥ypT3 and for those with ypN+, indicating that additional adjuvant therapy may be warranted to improve outcomes in this group. These patients met the current eligibility criteria for adjuvant nivolumab, supporting nivolumab as a reasonable and necessary option in this setting [11,20]. However, because the relevant subgroups—particularly pN+ UTUC and NAC-treated BC—were small, these observations should be interpreted as exploratory and potentially subject to type II error, rather than as definitive evidence of absence or presence of benefit in any given subgroup.

They also echo the broader perioperative landscape: in muscle-invasive bladder cancer (MIBC), cisplatin-based neoadjuvant chemotherapy (NAC) provides a modest yet durable survival advantage (~5% absolute 5-year OS; HR ~0.86) but its real-world use is constrained by cisplatin eligibility, and pathologic downstaging does not invariably translate into durable long-term control. Regarding upper tract urothelial carcinoma (UTUC), postoperative platinum combinations—not NAC—are the current evidence-based standard, determined by the strength of the randomized POUT trial findings, which showed a substantial disease-free survival benefit and a favorable trend in overall survival [1,5,7,9,21]. Once urothelial carcinoma recurs after definitive surgery, a cure is uncommon, and even after radical cystectomy, recurrences often arise within 2 years and long-term survival after relapse remains limited, underscoring the need to prevent recurrence where possible [1,22,23]. Recently, a potent first-line therapy for metastatic disease—enfortumab vedotin plus pembrolizumab (EV + P)—has shown high activity, including confirmed complete responses in ~29% of patients in the EV-302/KEYNOTE-A39trial; however, despite follow-up now approaching 2.5 years, long-term durability at the population level remains to be fully defined [10,11]. Accordingly, in bladder cancer, guideline-endorsed NAC followed by radical cystectomy has been adopted to lower the risk of relapse, with individual-patient meta-analyses demonstrating an ~5% absolute improvement in 5-year overall survival and ~9% improvement in 5-year disease-free survival versus surgery alone [1,4,7,24,25]. By contrast, UTUC is relatively rare—accounting for only ~5–10% of urothelial cancers—; therefore, adequately powered randomized trials have historically been difficult to conduct. POUT remains the key phase 3 evidence base, establishing adjuvant chemotherapy after nephroureterectomy as the standard of care and confirming durable disease-free survival gains on long-term follow-up [5,8,9].

In our BC analysis, among NAC-naïve patients, PFS and OS were favorable for cases of pT ≤ 2 but poorer for ≥pT3 and pN+ disease. When we evaluated the utility of AC across these strata (pT2, ≥pT3, and pN+), benefit was observed only in the pN+ subgroup, supporting a node-directed approach to postoperative cytotoxic therapy. Accordingly, if NAC is not administered, routine AC for pT2 may constitute overtreatment, whereas AC should be prioritized for pN+ disease. In the NAC-treated BC cohort, outcomes were likewise stage-dependent: PFS and OS were relatively favorable for ypT ≤ 2 (with a modest decrease in PFS for ypT2) but worse for ≥ypT3 and ypN+. Thus, among patients who receive NAC, the postoperative population most likely to derive adjuvant benefit appears to be those with ≥ypT3 and/or ypN+, aligning with current eligibility for adjuvant nivolumab. By contrast, although ypT2 without nodal involvement is label-eligible, routine adjuvant immunotherapy in this group may risk overtreatment and warrants careful shared decision-making regarding risks and benefits. Our data therefore suggest, but do not prove, that perioperative systemic therapy should be preferentially directed toward node-positive and ≥ypT3 subsets, whereas node-negative pT2/ypT2 disease may be more appropriately managed with observation and individualized consideration of adjuvant treatment.

From the perspective of adjuvant nivolumab eligibility, the NAC-treated BC cohort is particularly informative. In our series, seven patients (12.3%) had ypT2N0 disease after NAC. Under the CheckMate 274-based label, these patients would be considered high risk and eligible for adjuvant nivolumab on the basis of ypT2 stage alone. Yet, in our dataset, this ypT2N0 subgroup generally exhibited more favorable outcomes than ≥ypT3 or ypN+ disease, underscoring the possibility that uniform adjuvant immunotherapy in ypT2N0 could represent overtreatment for some patients. Although numbers are small and definitive conclusions cannot be drawn, these findings support a nuanced, shared decision-making approach that incorporates age, comorbidity, competing mortality, and patient preferences rather than relying solely on pathological stage.

In our UTUC analysis, among NAC-naïve patients, NUTRFS and OS were favorable for cases of pT ≤ 2 but poorer for ≥pT3 and pN+ disease. When we assessed the utility of AC across pT2, ≥pT3, and pN+ strata, benefit was observed for NUTRFS only in the pN+ subgroup, with no significant advantage for pT2 or ≥pT3 and no OS improvement in any subgroup. These findings suggest that efforts to improve OS in UTUC should focus on the ≥pT3 and pN+ risk groups—by optimizing NAC strategies or by deploying postoperative adjuvant immunotherapy (e.g., nivolumab) where appropriate [8,9,12]. However, clinical–pathologic staging discordance is substantial in UTUC—ureteroscopic biopsy-based and imaging-based clinical assessments (e.g., CT/MR urography) frequently under-stage tumors relative to final nephroureterectomy pathology; therefore, preoperative NAC may risk overtreatment in selected patients and warrants careful shared decision-making regarding risks and benefits [5,26,27,28,29,30,31]. Against this backdrop, our results reinforce a node-directed value proposition for AC across UC, while cautioning against routine use in node-negative, organ-confined BC. Given the small number of pN+ UTUC cases (*n* = 13), however, the apparent NUTRFS benefit of AC in this subgroup should be viewed as suggestive and in need of external validation rather than as definitive evidence.

Lymphovascular invasion (LVI) further contextualizes the risk: consistent with meta-analyses, LVI was associated with adverse outcomes in UC, and in our cohort its incidence varied by context—borderline higher in pN+ versus pT ≥ 2/pN− in BC (*p* = 0.056) but significantly higher in pN+ versus pT ≥ 2/pN− in UTUC (*p* = 0.012)—supporting LVI as a marker of biological aggressiveness [13,14,15,16]. Notably, however, analyses in BC have not consistently demonstrated a predictive interaction between LVI and AC benefit, underscoring the need for prospective validation [13,17]. The emergence of adjuvant nivolumab after radical surgery for high-risk UC (the CheckMate 274 trial) further elevates the importance of refined selection. Although the approved high-risk criteria (no NAC: pT3–4 and/or pN+; with NAC: ypT2–4 and/or ypN+) provide a pragmatic framework, our outcomes suggest that pT2/ypT2 without nodal involvement represents a lower-risk subset in whom routine adjuvant immunotherapy may constitute overtreatment when balanced against toxicity and competing risks [12]. Taken together, our data support prioritizing systemic therapy for node-positive disease, using LVI and related pathology to enrich the risk assessment, and pursuing prospective, biomarker-integrated strategies to validate LVI-anchored selection and to calibrate thresholds for adjuvant nivolumab—especially in pT2/ypT2—to avoid overtreatment while preserving meaningful benefit where the micrometastatic risk is greatest. At the same time, the degree to which LVI provides independent prognostic information beyond nodal status cannot be definitively established from our data, because multivariable analyses were limited by event numbers and potential overfitting; thus, the prognostic and predictive roles of LVI in the perioperative setting require prospective, centrally reviewed, biomarker-integrated evaluation.

An important consideration in interpreting our findings is the presence of selection bias and immortal time bias. Because treatment allocation (NAC/AC vs. observation) was not randomized, fitter and younger patients, or those with higher-risk pathological features, were more likely to receive perioperative chemotherapy, introducing confounding by indication. Furthermore, AC is administered after surgery; therefore, patients must survive and remain recurrence-free long enough to receive it. This “immortal time” can artificially favor AC groups in time-to-event analyses if not adequately accounted for. Although we attempted to mitigate bias through stage- and node-stratified analyses, residual confounding and immortal time bias cannot be excluded and may influence the magnitude and direction of observed associations.

This study had some limitations. Its retrospective, single-center design introduces selection bias and unmeasured confounding factors (e.g., performance status, renal function, and surgeon/pathologist variability) that might have influenced AC selection, dose intensity, and outcomes. The long accrual period (1998–2021) spans changes in imaging, pathological processing (including LVI ascertainment), perioperative care, and systemic regimens, potentially introducing temporal heterogeneity. We considered sensitivity analyses stratified by calendar period to explore temporal effects (e.g., pre- vs. post-GC era), subgroup sizes and event counts were insufficient to support robust conclusions. Pathology was not reviewed centrally, and LVI assessment was subject to interobserver variability; missing or incomplete data also necessitated exclusions. These real-world features reflect typical clinical practice but may reduce the precision and reproducibility of our estimates. Subgroup sizes—particularly NAC-treated BC and pN+ UTUC—were relatively small, limiting the power for interaction testing. Because AC is delivered postoperatively, immortal time and indication biases are possible despite time-to-event methods. We did not perform fully adjusted multivariable Cox proportional hazards models including LVI, pT stage, and pN status in the entire cohort because of concerns about model instability and overfitting given the limited number of events in several subgroups; accordingly, the independence of LVI as a prognostic factor relative to nodal status remains uncertain in this dataset. Finally, we did not incorporate molecular classifiers or circulating tumor DNA, which may refine residual risk estimation and guide treatment selection. Treatment-related adverse events, dose reductions, and treatment completion rates were also not systematically captured, limiting our ability to balance efficacy against toxicity and to fully define the risk–benefit profile of perioperative chemotherapy in this population. These limitations underscore that our findings should be considered hypothesis-generating and warrant confirmation in prospective, biomarker-integrated cohorts.

## 5. Conclusions

Perioperative benefit in UC was node-dependent: AC benefited pN+ disease but not node-negative, organ-confined pT2/ypT2. After receiving NAC, the risk of recurrence was clustered in ≥ypT3/ypN+, supporting adjuvant strategies for these groups. LVI enriched the risk but was not reliably predictive; thus, prospective, biomarker-integrated validation is warranted. Overall, our data suggest—but do not definitively establish—that a node-directed perioperative strategy may help to avoid overtreatment in lower-risk pT2/ypT2N0 disease while focusing systemic therapy on patients at the greatest risk of micrometastatic relapse. Future prospective trials incorporating standardized pathology, contemporary systemic regimens, and molecular biomarkers will be essential to validate and refine these observations.

## Data Availability

The data that support the findings of this study are available from the corresponding author upon reasonable request.

## Abbreviations

The following abbreviations are used in this manuscript:

AC	adjuvant chemotherapy
BC	bladder cancer
LVI	lymphovascular invasion
MIBC	muscle-invasive bladder cancer
NAC	neoadjuvant chemotherapy
NUTRFS	non-urinary tract recurrence-free survival
OS	overall survival
PFS	progression-free survival
RC	radical cystectomy
RNU	radical nephroureterectomy
UC	urothelial carcinoma
UTUC	upper tract urothelial carcinoma
ypT	pathological T stage after neoadjuvant therapy
ypN	pathological nodal status after neoadjuvant therapy
